# Cognition and Cognitive Training in Patients with Neurodegenerative Diseases

**DOI:** 10.3390/brainsci15020183

**Published:** 2025-02-12

**Authors:** Marco Cavallo, Stefano Lasaponara

**Affiliations:** 1 Department of Theoretical and Applied Sciences, eCampus University, 22060 Novedrate, Italy; 2 Department of Psychology, “Sapienza” University of Rome, 00185 Rome, Italy; stefano.lasaponara@uniroma1.it

## 1. Introduction

This Special Issue presents some recent developments in the intriguing research and clinical field of cognition and cognitive training in patients with neurodegenerative diseases. It aims to stimulate interest in this area and encourage the further exploration of effective evidence-based treatment strategies. Neurodegenerative diseases, such as Alzheimer’s disease (AD), Parkinson’s disease (PD), frontotemporal dementia (FTD), and others, typically lead to progressive cognitive decline, mainly affecting the realms of memory, executive function, attention, and language. The cognitive deterioration significantly reduces patients’ and caregivers’ quality of life, often leading to early institutionalization. Despite a huge amount of research on the underlying pathophysiology of these disorders, there is a substantial lack of knowledge about how to detect and manage the progression of cognitive deficits appropriately.

## 2. Recent Developments in Cognitive Training for Neurodegenerative Diseases

Over the last decade, there has been increasing interest in non-pharmacological interventions, such as cognitive training, designed to maintain, as much as possible, an acceptable level of ‘cognitive health’ in patients affected by neurodegenerative conditions. The term ‘cognitive training’ refers to exercises that enhance specific cognitive skills, such as memory, attention, processing speed, and executive functions. Various approaches have been developed, including paper-and-pencil protocols, computerized cognitive training, and even recent Virtual Reality interventions. Most of these approaches often include exercises targeting the cognitive domains that are often affected in the early stages of the particular clinical condition.

The literature in the field shows mixed results regarding the effectiveness of cognitive training with these clinical targets. For example, in patients with MCI, computerized cognitive training programs targeting memory and executive function have demonstrated small to moderate improvements in cognitive performance [1], particularly when implemented in the early stages of the condition. These results are supported by studies suggesting that cognitive training may have a neuroplasticity-promoting effect, which could help delay cognitive decline by enhancing the brain’s ability to reorganize and compensate for neuronal loss. Similarly, patients with PD, who often experience cognitive deficits in the realms of executive function, attention, and memory [2], have also been targeted by cognitive training interventions [3]. Some studies have indicated improvements in cognitive performance after training, especially when the interventions were combined with physical exercises to promote both cognitive and motor function. The potential synergistic effects of integrated cognitive and physical training are being actively explored and offer a promising avenue for future research. A recent development consists of using technology to deliver cognitive training (e.g., via mobile apps or Virtual Reality equipment). These systems provide an engaging way to deliver tailored cognitive exercises to patients and make monitoring the training’s progress relatively easy. While the application of technology in this field is still in its infancy, the potential to expand the upscaling of ‘high-tech’ cognitive training is a prospect that deserves attention.

However, despite these promising findings, several key challenges remain. One key issue is the limited understanding of the specific mechanisms through which cognitive training exerts its effects. While it has been reported that cognitive training may enhance brain plasticity (see, for example, [4]), reduce cognitive decline, or even slow the progression of the disease, these claims have not been consistently replicated across different populations or disease stages. Secondly, the optimal parameters for cognitive training—such as duration, intensity, and type of exercises—are not yet well established. Additionally, patient heterogeneity presents a significant challenge. Even if it is convenient for researchers to refer to, for instance, a “group of AD” or “PD patients”, neurodegenerative diseases manifest differently across individuals, not only in terms of clinical presentation, but also in terms of underlying pathology and rate of progression. This fundamental diversity in symptomatology suggests that a “one-size-fits-all” approach may not be promising. While it seems important to tailor cognitive training to the patient’s needs, there is a lack of a clear consensus on how best to do it. Another critical issue lies in understanding how cognitive training interacts with pharmacological treatments. Many patients affected by neurodegenerative diseases are treated with multiple medications that have an impact on symptomatic cognitive dysfunction, such as cholinesterase inhibitors in AD or dopaminergic agents in PD. How these medications may interact with cognitive training and whether combined treatments lead (or not) to more favorable outcomes remain largely unexplored issues. Lastly, while cognitive improvements following training have been consistently reported (see, for instance, [1,5]), it is still unclear whether these improvements translate into meaningful functional benefits in patients’ daily lives and whether these improvements are stable over time once the training has been interrupted. Cognitive function and performance on standardized cognitive tests are not significantly correlated with real-world abilities, such as managing finances, engaging in social interactions, or performing daily living activities. As such, future research must emphasize the need to examine the real-world impact of cognitive training on patients’ functional abilities and, ultimately, on their quality of life.

## 3. Overview of the Five Selected Contributions

The five empirical articles included in the present Special Issue tackle the relevant aspects of this topic and provide interesting preliminary evidence. The first contribution (by Areti Batzikosta et al.) explores the relationship between specific Theory of Mind (ToM) dimensions, cognitive planning, and sleep duration in subjects with mild cognitive impairment (MCI) and healthy controls (HC). The authors found that the HC group performed better than the amnestic and non-amnestic MCI groups in ToM tasks and that the amount of sleep had significant indirect effects through cognitive planning on specific ToM aspects. Their findings highlight the importance of considering the relationship between the ToM deficits associated with MCI and decreased sleep duration.

The second contribution (by Fonte et al.) focuses on the combined effect of transcranial Direct Current Stimulation (tDCS) and motor or cognitive activity in patients with AD. Interestingly, the authors report significant differences between anodic tDCS groups and sham tDCS groups, with improved global cognitive status, selective attention, and sustained attention in the former. No significant differences between the two anodic tDCS groups before and after treatment were found. Their findings suggest that combined anodal tDCS with motor or cognitive activity could positively impact at least some cognitive functions. However, more robust evidence is necessary to corroborate this intriguing hypothesis.

The third contribution (by Morganti et al.) adopts a different perspective and focuses on AD patients’ caregivers. The authors assess the effectiveness of a psychoeducational program based on a Virtual Reality (VR) application that presents users with typical ecological situations from the perspective of patients with dementia. The study involves informal caregivers of individuals with AD who were asked to participate in a six-session psychoeducation program. The immersive VR sessions allowed for the participants to experience first-hand some frequent symptoms of dementia (e.g., spatial disorientation, difficulty in problem-solving, and anomia) in “everyday life” settings. Their results indicate that VR shows efficacy in producing a sense of first-person participation in caregivers, although further research is clearly needed to corroborate this scientific intuition.

The fourth contribution (again by Areti Batzikosta et al.) is strictly related to the topic already investigated by this research group (the association between cognitive control abilities and sleep parameters in MCI). Regarding objective sleep measurements, total sleep time (TST) was measured using actigraphy, indicating significant differences between groups, with the healthy control group having a significantly higher mean TST than the MCI group. Interestingly, the authors report a significant association between TST and performance using a cognitive measure (Tower Test). The study’s preliminary findings suggest potentially valuable parameters supporting the connection between sleep duration and cognitive planning measures.

Lastly, the fifth contribution (by Stanzani-Maserati et al.) represents an ambitious attempt to investigate the cognitive domain through a projective personality tool (the Village Test). In total, 50 patients with AD, 28 patients with MCI, and 38 controls were included in this study. The AD group built smaller and more essential villages with reduced use of pieces, limited dynamic pieces, and low use of human figures. According to the authors, the villages built by the AD group may represent a cognitive and affective coarctation and indicate a sense of disorientation and isolation. In their view, this test may be considered a helpful tool to study the inner emotional and existential states of patients with AD, and it could represent a complementary screening tool for orienting clinical diagnosis and intervention.

## 4. Conclusions

The field of cognitive training in neurodegenerative conditions has received growing attention in recent years, with various studies demonstrating the potential benefits of non-pharmacological interventions, while others demonstrate otherwise. The literature published in the last 20 years, alongside the recent contributions included in this Special Issue, have provided interesting preliminary evidence on this crucial clinical topic. An essential issue pertains to detecting the ‘ecological’ impact of cognitive training to design and implement more effective and personalized interventions to improve cognition and the quality of life of patients and their caregivers. We hope that cognitive training may soon become a ‘cornerstone’ of care for patients affected by neurodegenerative conditions, helping them achieve the best possible outcome in the unequal fight against their disease.
